# Effect of the time scale on the uncertainty of geometric mean concentrations of fecal indicators in creek under baseflow conditions

**DOI:** 10.1038/s41598-020-58603-5

**Published:** 2020-02-03

**Authors:** Dong Jin Jeon, Yakov Pachepsky, M. Dana Harriger, Rachael Zhu, Cary Coppock

**Affiliations:** 1 USDA-ARS, Environmental Microbial and Food Safety Laboratory, Beltsville, Maryland USA; 2 Korea Environment Institute, Devision for Integrated Water Management, Sejong, Korea; 3 Wilson College, Division of Integrated Sciences, Chambersburg, Pennsylvania USA

**Keywords:** Freshwater ecology, Microbial ecology, Limnology

## Abstract

Geometric mean concentrations of fecal indicator bacteria *E. coli* and enterococci are commonly used to evaluate the microbial quality of irrigation, recreation, and other types of waters, as well in watershed-scale microbial water quality modeling. It is not known how the uncertainty of those geometric mean concentrations depends on the time period between sampling. We analyzed data collected under baseflow conditions from three years of weekly and several daily sampling campaigns at Conococheague Creek in Pennsylvania. Standard deviations of logarithms of geometric mean concentrations were computed over weeks, months, and seasons. The increase in standard deviations from weekly to seasonal time scale was on average about 0.1 and 0.2 for log(*E. coli*) and log(enterococci), respectively, and in most cases was statistically significant. This may need to be accounted for when evaluating the uncertainty of measurements for modeling purposes and in risk assessment of microbial water quality.

## Introduction

Presence of fecally-derived bacteria in water indicates a potential human health risk due to direct contact with water during recreational activities or eating produce that was irrigated or washed using the water^1–3^. The U. S. Environmental Protection Agency (EPA) is currently using and the U. S. Food and Drug Administration (FDA) has suggested to use generic *Escherichia coli* (*E. coli*) and enterococci as indicators of fecal contamination for recreational water and irrigation water^4^. Both regulatory agencies use the geometric mean of those indicator bacteria concentrations. However, they use different time scales. The EPA uses the month time scale, and geometric mean is computed over four to five weekly observations. The FDA has suggested taking 20 concentration measurements over a longer evaluation period of two to four years, with the annual number of measurements being between five and ten. In such a case the time scale is the produce vegetation period. There also is the week time scale which is of interest if one uses monitoring data in watershed-scale water quality modeling. The uncertainty of a single day measurement reported as the logarithm of concentration can be estimated from the uncertainty in the geometric mean of concentrations during a week encompassing the observation day (i.e., Hong *et al*.^5^). Thus, knowing the uncertainty of the geometric means appears to be important for estimating risks of fecal concentrations exceeding the threshold values set in microbial water quality standards, and for comparing the uncertainty of model predictions with the uncertainty in data. The scale of interest for the concentration geometric mean uncertainty depends on the intended use of concentration measurements. Large creeks and rivers typically have multiple uses, and therefore estimates of the geometric means of indicator bacteria concentrations at different scales are needed.

Although studies of the uncertainty of geometric means of indicator bacteria concentrations have a substantial history in the research addressing health issues (i.e. Gronewold *et al*.^6^; Heberger *et al*.^7^), no data are available on the effect of time scale over which the geometric mean was computed on the uncertainty in the value of this geometric mean. The work of Muirhead and Meenken^8^ presents the only known to us exemption; the authors examined the differences of temporal variability under baseflow condition at different relatively short time scales (minutes, hours, and days) and observed the increase in variability with the increase of time scale.

The objective of this work was to evaluate and compare the temporal variability of the geometric mean concentrations of *E. coli* and enterococci in a creek at weekly, monthly, and seasonal time scales for the intensively monitored large creek in Pennsylvania with multiple water uses.

## Results and Discussion

**Figure 1:**
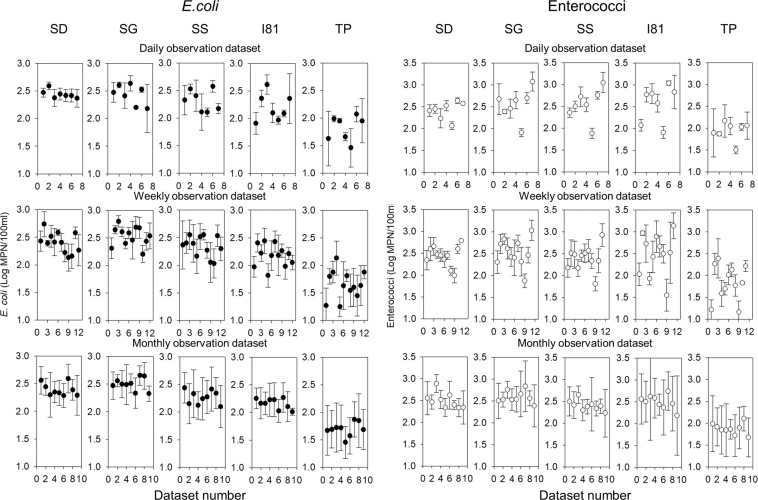
Geometric mean and standard deviation values of logarithms of *E. coli* and enterococci concentrations for daily, weekly, and monthly observation datasets at monitoring locations TP, I81, SS, SD, and SG.

Figure 1 shows the mean and the standard deviation values of logarithms of daily, weekly and monthly *E. coli* and enterococci concentrations on different sampling times and locations. The datasets were numbered in the sequence they were extracted from the concentration time series. The mean values of logarithms of *E. coli* concentrations tended to increase downstream. The mean values for the same time scale varied greatly with the measurement period at daily and weekly datasets (Fig. 1). The monthly dataset had a relatively lower variation in the mean values. The mean values of enterococci concentration were substantially lower at TP location compared to the other locations at all time scales. Similar mean values across locations were observed in the monthly datasets.

The supplementary Table 1 shows the probabilities of median logarithms of concentrations being the same in pairs of locations. There were very low (P < 0.001) probabilities that median logarithm concentrations at TP are the same as at any other observation location. Also, low probabilities (<0.01) were found for the median logarithms of *E. coli* concentrations to be the same in pairs of locations I81-SD and I81-SG. For other location pairs, probabilities of having the same median logarithm *E. coli* concentrations were larger than 0.01 but remained mostly low. Weekly observations in location pair SD-SG presented the exception, the median logarithm concentrations in these locations were the same with a probability of 0.97. For enterococci, only TP paired with other locations showed a low probability of median concentrations being the same. Larger than 0.1 values of probabilities of having the same median logarithm of enterococci concentration were found for all other pairs of locations. Overall, the enterococci dataset appeared to be more homogeneous along the creek. It is possible that changes in land use along the creek from forest to agricultural use to mixed suburban and agricultural use had less influence on the downstream trend of enterococci.

**Figure 2:**
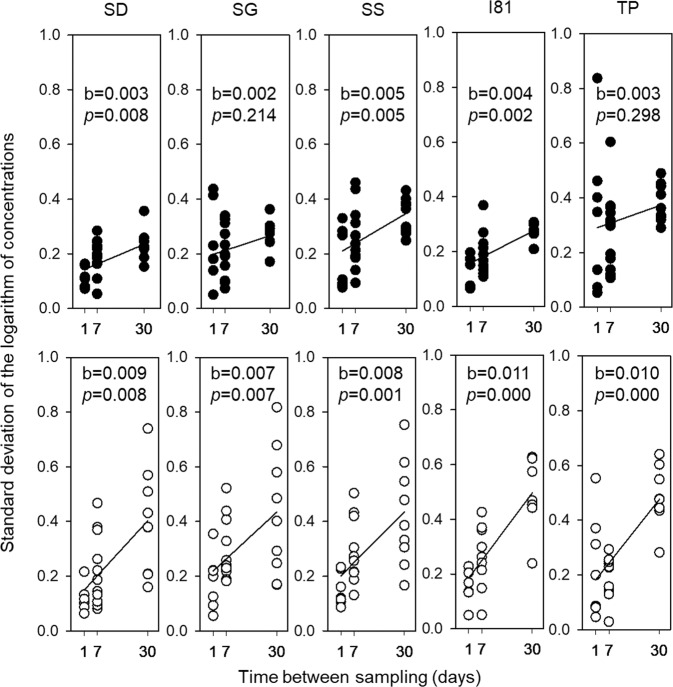
The distribution of standard deviation values and regression slopes of logarithms of *E. coli* (filled circles) and enterococci concentrations (hollowcircle) according to the different time scales at five monitoring locations. b and p are the slope value and p-value for the regression slope test.

Standard deviations of the logarithms of indicator concentrations are shown in Fig. 2. The maximum values of the standard deviations were larger for enterococci than for *E. coli* in all locations for monthly observations. The standard deviations across monitoring locations were mostly not significantly different at the same time scales both for *E. coli* and enterococci. The TP location presented an exemption, standard deviations were significantly different from other locations except for the SS. Overall, enterococci demonstrated higher variability. Differences between the variability of *E. coli* and enterococci concentrations may indicate that they are coming to the stream from different sources. These differences may also manifest the differences in survival of the two organisms in various environmental compartments.

The trend of the increase of the standard deviations with time scale was found for both indicator organisms at all locations (Fig. 2), after a total of 6% and 5% values of standard deviations of logarithms of concentrations were removed as outliers from *E. coli* and enterococci datasets, respectively. Linear regression equations were developed to quantify this trend. The independent variable was the time between sampling, i.e. 1, 7, and 30 days for weekly, monthly and seasonal time scales respectively. Slopes for *E. coli* were significantly different from zero at SD, SG and TP locations. The probabilities of slopes being zero at SS and TP locations were larger than 0.05 but still low. Slopes for enterococci were significantly different from zero at all locations.

The slope for *E. coli* at the TP location was significantly different from other locations except for the SS location, and the slopes at SS and SD locations were not significantly different. The slopes for enterococci did not differ significantly among all the locations.

For the time scale increase from daily to seasonal, values of slopes indicated the increase in standard deviation on average by about 0.1 and 0.2 for log(*E. coli*) and log(enterococci), respectively (Fig. 2). Several factors might contribute to the increase of the uncertainty in geometric mean concentrations with time scale. Weekly and monthly measurement dates were separated by rainfall events of different intensity. Spatially and temporally variable rainfall events could increase variability in indicator bacteria inputs to water with runoff and from the bottom sediment^9–12^. Further increase of time between sampling could also involve changes in sources of fecal indicator bacteria between sampling such as grazing locations, manure application, and wildlife visits. It appears that increase in variability of the geometric mean concentrations with time scale is not large but can be statistically significant and may need to be accounted for if the uncertainty in the geometric mean of concentrations was estimated at the time scale different from the task at hand.

Knowing the uncertainty of geometric means allows one to assess risks of making the incorrect conclusion about the exceeding the regulatory standards on microbial water quality for a water source. It also allows one to define the number of datasets to determine the geometric mean concentration of indicator organisms with the preselected accuracy. Having the estimate of the uncertainty of daily concentration measurements can be useful for calibration of watershed-scale microbial water quality models, that are used, in particular, in mandated Total Maximum Daily Load determinations related to the microbial impairment of surface water sources^13^. Transferring information about the uncertainty between scales can allow for the more efficient use of existing data collected at specific scales, and for the design of feasible monitoring schedules with subsequent transfer of the variability metrics across the scales.

## Conclusions

A three-year-long weekly monitoring of the 20 km reach at the Conococheague Creek in Pennsylvania showed an increase of geometric mean concentrations of *E. coli* and enterococci along the creek as land use changed from forest to agricultural to mixed suburban and agricultural. Variability of logarithms of geometric mean concentrations of fecal indicator bacteria increased with the increase of the time interval between sampling. The increased rate for enterococci was about twice that of *E. coli*. The dependence of the fecal indicator variability on time scale can be statistically significant and may need to be accounted for in evaluating uncertainty measurements, and in risk assessment of microbial water quality for recreation and irrigation.

## Methods

The study was carried at the Conococheague Creek headwaters in Franklin County, south -central Pennsylvania. Five primary sampling sites were established where water samples were collected (Fig. S1). The TP site is the most upstream station, the sampling stations I81, SS, SG, and SD are established on 10.3 km, 13.6 km, 17 km, and 22.8 km from TP respectively to SD. TP and I81 drain the forested area and the agricultural areas, respectively, the other three sampling sites receive both agricultural and urban inputs.

Water samples were collected from the five sites for *E. coli* and enterococci. Weekly water samples were collected over three years from October 2015 to October 2018 in the morning hours. In July and August of 2017 and 2018, samples were collected daily (Monday through Friday) on alternating weeks. The total number of sampling days was 179. Environmental grab samples (1 liter) were drawn and serially diluted with peptone buffer solution. They were further vacuum filtered using 45 µm filters. *E. coli* filters were plated and incubated on the membrane using thermotolerant *E. coli* agar (mTEC) at 35 °C for two hours and 45 °C for 22–24 hours. The second subsample was incubated for enterococci using mEI agar for 24 hours at 41 °C.

To analyze the variability of microbial concentrations at tdifferent time scales in baseflow conditions during the vegetation (May to October) period, some water monitoring data were excluded based on the estimated duration of surface runoff, and rainfall threshold exceedance. The duration of the surface runoff was estimated using the empirical equation derived by Linsley *et al*.^14^ in the geographically close catchment as follows:

1 $${\rm{N}}={{\rm{A}}}^{0.2}$$

where N is the number of days after which surface runoff ceases, and A is the catchment area in square miles. The estimated duration of surface runoff varied from 2 days at the TP site to 2.5 days at the SD site. The 10 mm threshold was used to define significance of rainfalls as suggested by Han *et al*.^15^ for the northern subtropical climate in similar land use and weather conditions similar to those in Han *et al*.^15^ work. Thus, we included data from the sampling days that had the cumulative precipitation lower than 10 mm during three preceding days from May to October.

Standard deviations of logarithms of *E. coli* and enterococci concentrations were computed (a) over daily observations on the weeks when such observations were available, (b) over four consecutive weekly observations, and (c) over consecutive monthly observations on the same week of each month. These values characterized the variability of daily, weekly, and monthly observations, respectively. Statistical analysis was conducted using PAST^16^ software and the R software. Kruskal-Wallis test was used to test the hypothesis that the samples are taken from populations with equal medians. The Dunn’s post-hoc test verified that medians of logarithms of concentrations at pairs of locations were significantly different. The boxplot method^16^ was used to remove outliers in standard deviations of logarithms datasets. Analysis of covariance was performed to compare the homogeneity of regression slopes among the monitoring locations followed by a Tukey post hoc test.

### Disclaimer

Mention of trade names or commercial products in this publication is solely for the purpose of providing specific information and does not imply recommendation or endorsement by the U.S. Department of Agriculture. USDA is an equal opportunity provider and employer.

## Supplementary information

Supplementary information.
