# Supplementary material for: Effect of the time scale on the uncertainty of geometric mean concentrations of fecal indicators in creek under baseflow conditions
Source: Sci Rep. 2020 Feb 3;10:1720. doi: 10.1038/s41598-020-58603-5 (PMC6997410; doi:10.1038/s41598-020-58603-5)
Supplement: Supplementary file 1 — Supplementary information. [file 41598_2020_58603_MOESM1_ESM.docx]

**Effect of the time scale on the uncertainty of geometric mean concentrations of fecal indicators in creek under baseflow conditions**

Dong Jin Jeon^1,2*^, Yakov Pachepsky^1^, M. Dana Harriger^3^, Rachael Zhu^3^, Cary Coppock^1^

^1^USDA-ARS, Environmental Microbial and Food Safety Laboratory, Beltsville, Maryland, USA

^2^Korea Environment Institute, Devision for Integrated Water Management, Sejong, Korea

^3^Wilson College, Division of Integrated Sciences, Chambersburg, Pennsylvania, USA

*djjeon@kei.re.kr

Figure S1. Land use map of the Conococheague Creek watershed and monitoring locations.


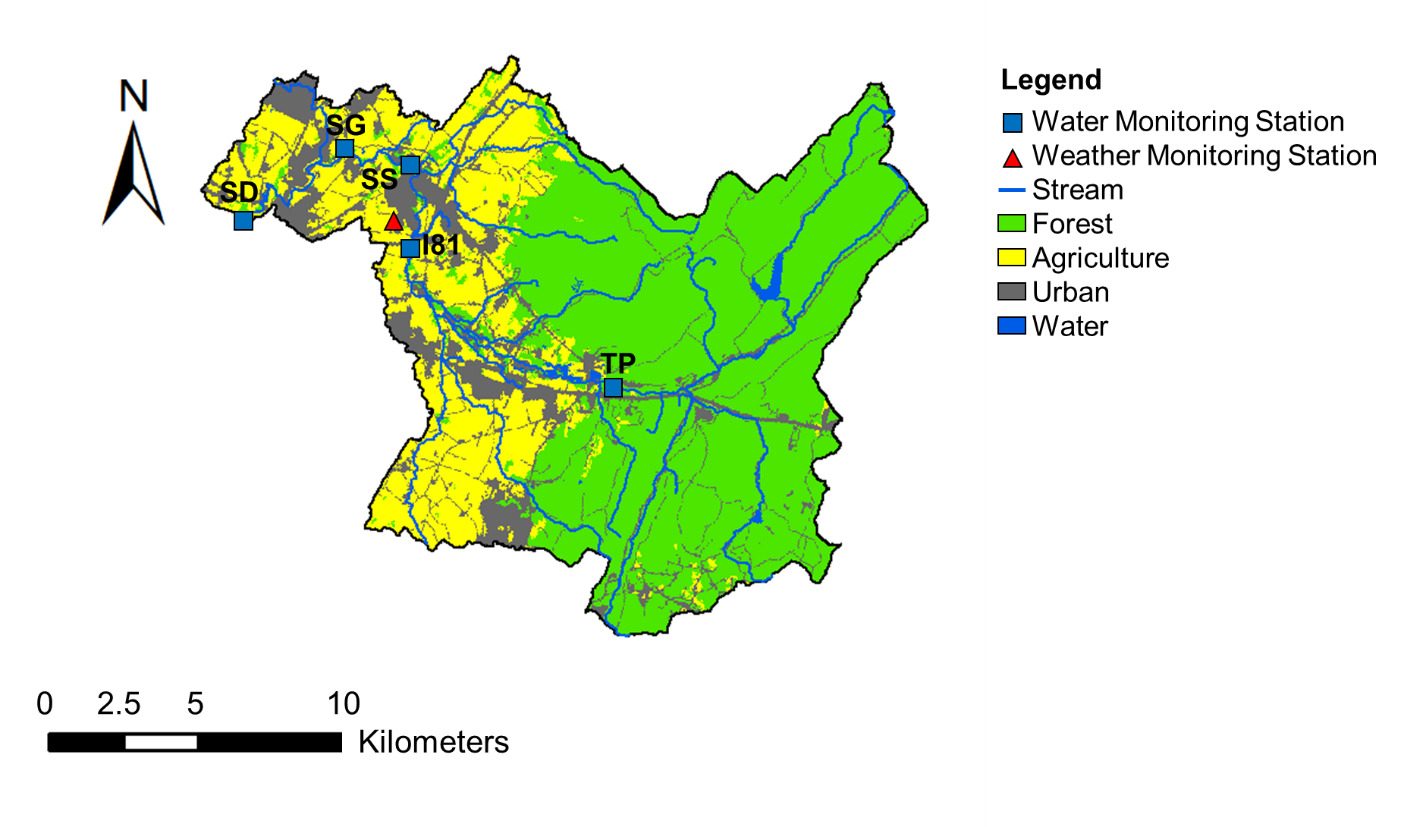


Table S1. *p*-values from Dunn’s post-hoc test for comparing medians of logarithms of *E. coli* and enterococci concentrations at pairs of locations.

|  |  | *E. coli* | | | | |
| --- | --- | --- | --- | --- | --- | --- |
|  |  | SD | SG | SS | I81 | TP |
|  |  | Daily measurements | | | | |
| Enterococci | SD |  | 0.967 | 0.136 | 0.006 | 0.000 |
|  | SG | 0.287 |  | 0.143 | 0.007 | 0.000 |
|  | SS | 0.371 | 0.864 |  | 0.209 | 0.000 |
|  | I81 | 0.285 | 0.997 | 0.861 |  | 0.000 |
|  | TP | 0.000 | 0.000 | 0.000 | 0.000 |  |
|  |  | Weekly measurements | | | | |
|  | SD |  | 0.951 | 0.337 | 0.001 | 0.000 |
|  | SG | 0.369 |  | 0.008 | 0.000 | 0.000 |
|  | SS | 0.494 | 0.114 |  | 0.020 | 0.000 |
|  | I81 | 0.771 | 0.544 | 0.33 |  | 0.006 |
|  | TP | 0.000 | 0.000 | 0.000 | 0.000 |  |
|  |  | Monthly measurements | | | | |
|  | SD |  | 0.212 | 0.121 | 0.000 | 0.000 |
|  | SG | 0.337 |  | 0.005 | 0.000 | 0.000 |
|  | SS | 0.443 | 0.084 |  | 0.064 | 0.000 |
|  | I81 | 0.719 | 0.553 | 0.261 |  | 0.000 |
|  | TP | 0.000 | 0.000 | 0.000 | 0.000 |  |
